# Optimistic update bias holds firm: Three tests of robustness following Shah et al.

**DOI:** 10.1016/j.concog.2016.10.013

**Published:** 2017-04

**Authors:** Neil Garrett, Tali Sharot

**Affiliations:** Affective Brain Lab, Department of Experimental Psychology, University College London, London, UK

## Abstract

A diverse body of research has demonstrated that people update their beliefs to a greater extent when receiving good news compared to bad news. Recently, a paper by Shah et al. claimed that this asymmetry does not exist. Here we carefully examine the experiments and simulations described in Shah et al. and follow their analytic approach on our data sets. After correcting for confounds we identify in the experiments of Shah et al., an optimistic update bias for positive life events is revealed. Contrary to claims made by Shah et al., we observe that participants update their beliefs in a more Bayesian manner after receiving good news than bad. Finally, we show that the parameters Shah et al. pre-selected for simulations are at odds with participants’ data, making these simulations irrelevant to the question asked. Together this report makes a strong case for a true optimistic asymmetry in belief updating.

## Highlights

• Three tests reveal the robustness of optimistic belief updating.
• Optimistic belief updating is observed for both positive and negative life events.
• Belief updating is more Bayesian in response to good news than bad.
• It has been claimed that optimistic belief updating does not exist.
• We show that the methodologies used to make this claim are flawed.

## 1 Introduction

Numerous studies spanning behavioural economics (Eil and Rao, 2011, Krieger et al., 2016, Krieger et al., 2014, Möbius et al., 2012), psychology (Garrett and Sharot, 2014, Kuzmanovic et al., 2015, Moutsiana et al., 2013) and neuroscience (Garrett et al., 2014, Korn et al., 2012, Kuzmanovic et al., 2016, Lefebvre et al., 2016, Ma et al., 2016, Moutsiana et al., 2015, Sharot, Guitart-Masip et al., 2012, Sharot et al., 2011, Sharot, Kanai et al., 2012) have shown that people alter their beliefs to a greater extent in response to good news than bad news. This asymmetry can lead to a positive bias in beliefs regarding oneself, referred to as the *superiority illusion* (Hoorens, 1993, Kruger and Dunning, 1999, Svenson, 1981), and in beliefs regarding one’s future, referred to as *unrealistic optimism* (Calderon, 1993, Radcliffe and Klein, 2002, Shepperd et al., 2005, Weinstein, 1980, for review see Sharot & Garrett, 2016). The latter phenomenon, first described by Neil Weinstein (Weinstein, 1980), has since been supported by a large body of evidence (Armor and Taylor, 2002, Regan et al., 1995, Shepperd et al., 2003, Shepperd et al., 2013, Weinstein, 1987; for a review see: Sharot, 2011, Sharot, 2012).

A recent study by Shah, Harris, Hahn, Catmur, and Bird (2016) has revisited the phenomenon of optimistic asymmetry in belief updating. The authors claim that an optimistic bias is unlikely, apart from very specific cases including sports fans and smokers. Whilst Shah et al. present empirical evidence from a number of experiments, findings across their studies are inconsistent and methodological issues lie at the heart of their experimental design. Nonetheless, the research questions they attempt to address are important and each constitute interesting tests of the robustness of optimistic updating. In this report, we carefully examine the work and reveal statistical, methodological and conceptual problems, which when corrected supports the robustness of the asymmetry in belief updating.

In particular, the authors of that paper: (i) claim that not taking into account subjects’ beliefs regarding base rates can lead to misclassification of trials, which causes the update bias; (ii) claim to show biased updating in Bayesian agents; (iii) claim to empirically show pessimistically biased updating for positive life events. Whilst the report may sound compelling at first read, an informed examination of the evidence casts significant doubts on these claims.

Specifically, in this report we detail that: (i) previous studies have shown that after taking into account subjects’ base rates, exactly as Shah et al. lobby for, an optimism update bias is still observed (Garrett and Sharot, 2014, Kuzmanovic et al., 2015); (ii) comparing human data to Bayesian agents shows that subjects are less Bayesian when updating their beliefs in response to bad news than good news, thus the optimistic update bias cannot be explained away as Bayesian. Shah et al.’s simulations of Bayesian agents rest on specific parameters that are incompatible with human data; (iii) Shah et al.’s finding of a pessimistic update bias for positive events is due to a confound in the set of stimuli they used. When avoiding this confound no pessimistic update bias is observed for positive events.

### 1.1 Misclassification argument is of no empirical consequence

In their paper (Shah et al., 2016), Shah et al. outline an issue that has been described and resolved in two previous papers (Garrett and Sharot, 2014, Kuzmanovic et al., 2015). The basic claim (outlined previously in Garrett & Sharot, 2014) is that participants may hold one estimate regarding their own likelihood of experiencing an event and another regarding the likelihood for someone like them. Participants then receive information regarding the likelihood of “someone like them” to experience a specific event. Trials are classified according to whether the information is better or worse than the participant’s estimate of their own likelihood of experiencing that event. One can imagine a situation where a participant believes their own likelihood of being robbed, for example, is 10% but believes the likelihood for someone like them is 40%. They then learn that the likelihood for someone like them is in fact 30%. That trial will then be classified as a “bad news” trial (because 30% is worse than 10%) when in fact it should be classified as a “good news” trial (because 30% is better than 40%).

**Fig. 1:**
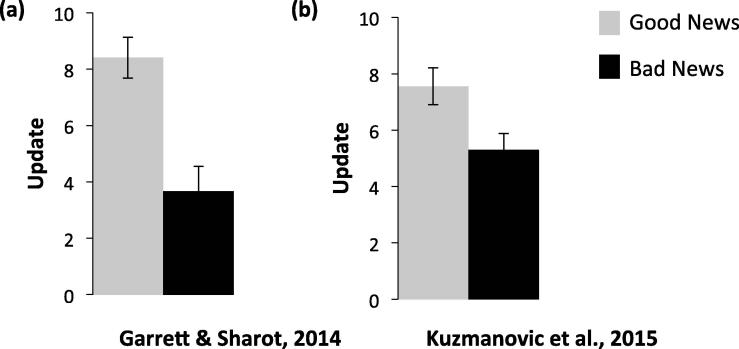
Update bias under alternative classification scheme. Two independent studies have shown that when trials are categorized according to participants’ estimates of base rates (as advocated by Shah et al., 2016) an optimistic update bias remains. This has been shown in (a) Garrett and Sharot (2014) and (b) Kuzmanovic et al. (2015). *Error bars represent SEM.*

This concern has been empirically tested and resolved before (Garrett and Sharot, 2014, Kuzmanovic et al., 2015). In two previous studies, participants’ estimates of base rates were elicited in addition to their self-estimates. Trials were then classified according to whether provided base rates were lower or higher than participants’ estimated base rates.^1^ In both studies, an optimism update bias persists even after this classification method is applied (Fig. 1). This is because only a small number of trials are in fact misclassified (Garrett & Sharot, 2014).

### 1.2 Optimistic update bias cannot be explained away as Bayesian

**Fig. 2:**
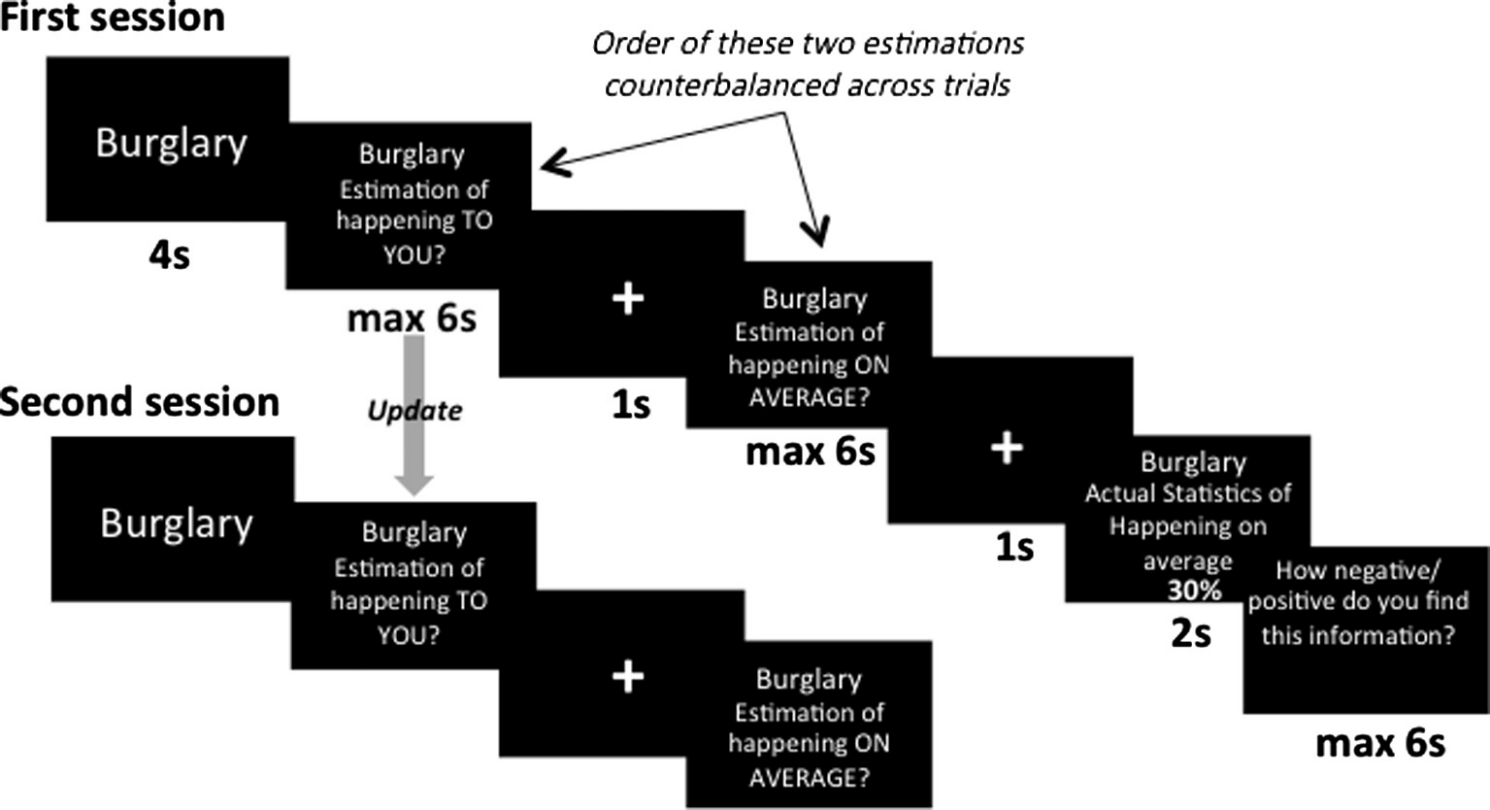
Belief update paradigm used in Garrett and Sharot (2014). On each trial, participants were presented with a short description of 1 of 80 adverse events and asked to estimate how likely this event was to occur to themselves in the future and how likely the event was to happen on average in the population. They were then presented with the average probability of that event occurring in a demographically similar population. Finally, participants were asked to rate how negative/positive they found this information. The second session was the same as the first except that the average probability of the event to occur was not presented and participants did not submit any ratings.

Previous studies have found that subjects are less Bayesian in updating their beliefs when receiving bad news than good news, consistent with the idea that asymmetric updating is not purely Bayesian (Eil and Rao, 2011, Möbius et al., 2012). Shah et al. test this same question using the belief update task for future life events. Their findings, across four studies, were inconclusive. We thus employed the same analysis carried out by Shah et al. on our previously published set of data from 32 participants (Garrett & Sharot, 2014), also classifying trials as advocated by Shah et al. (see Section 1.1). Our Materials and Methods have been reported in detail elsewhere (Garrett & Sharot, 2014) and we summarize them in Fig. 2. The calculations advocated by Shah et al. (2016), were used and summarized in Box 1.

**Fig. 3:**
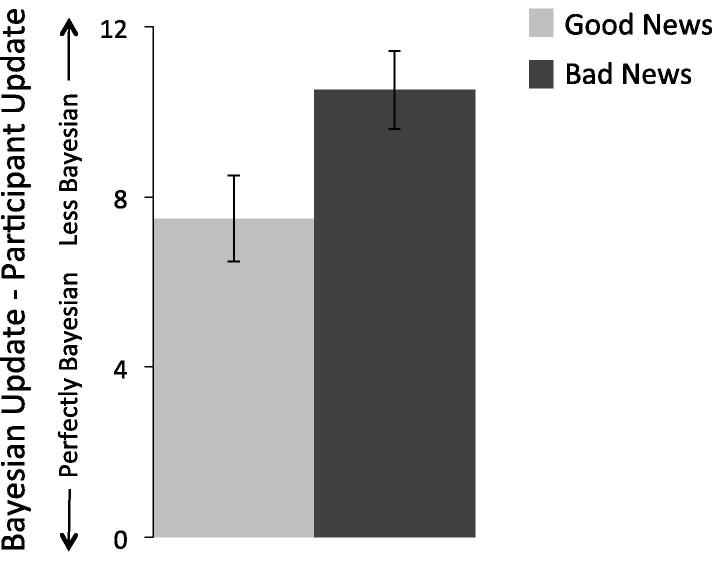
Updating compared to rational Bayesian agents. Participants’ update in a less Bayesian manner in response to bad news than good news. Scores indicate the difference between a Bayesian agent and participants’ update. The classification method and analysis championed by Shah et al. was used. Small numbers indicate more normative updating (i.e. closer to a Bayesian agent). This new analysis uses data from Garrett and Sharot (2014). *Error bars represent SEM.*

We found that comparing participants updates to that of a perfectly rational, Bayesian agent revealed that participants were less likely to update their beliefs in a Bayesian manner in response to bad news than good news (Fig. 3 shows the new analysis of previously published data (from Garrett & Sharot, 2014): t(31) = 2.53, p < 0.05, paired sample *t*-test). Similar findings, using the belief update task, were recently reported by Kuzmanovic and Rigoux (2016). Contrary to the claim of Shah et al., these results, as well as past ones (Eil and Rao, 2011, Möbius et al., 2012), support a true valence-dependent asymmetry in how humans update beliefs.

Shah et al. also conduct a simulation using hypothetical data that consists of one specific base rate (30%) and 52 hypothetical agents, 93% of which are fixed to have likelihood ratios (LHR) less than 1. Likelihood ratios express how diagnostic the evidence available to a participant is. Shah et al. (2016), claim to show an optimistic update bias in the agents used in their simulation. However, the authors themselves note that if the likelihood ratios are greater than 1, the opposite pattern ought to ensue - greater updating for bad news compared to good news. *If likelihood ratios are close to 1, no bias will emerge in either direction in Bayesian agents*. This raises the question: what are the likelihood ratios that human participants use in this task?

Using the same set of data we reported above (published previously in Garrett & Sharot, 2014), we found that the mean likelihood ratio across participants was 1.12 (s.d.: 0.43), which was not significantly different than 1: t(32) = 1.63, p = 0.11, one sample *t*-test against test value of 1. A separate data set from an independent research group found that likelihood ratios were 1.55 on average (Kuzmanovic & Rigoux, 2016). Despite having the empirical data to derive likelihood ratios from their own experiments, Shah et al. select to fix the vast majority of likelihood ratios in their simulation to those that are less than 1 (mean likelihood ratio = 0.70, significantly less than 1: t(51) = 9.66, p < 0.001, one sample ttest against test value of 1). This assumption, however, is not supported by the data. These simulations are thus irrelevant to the question of whether the optimistic update bias observed in participants is Bayesian.^2^

It is worth noting that following the equation in Box 1, on each individual trial the value of the LHR (less than 1, equal to 1, larger than 1) indicates whether a subject believes their own risk is lower/equal/higher than someone like them. However, this logic does not hold for average estimates. That is to say, it is mathematically possible for the average risk estimates of a subject to be lower for themselves than others, yet for the average LHR to be equal or higher than 1. This is indeed the case in Garrett and Sharot (2014).

### 1.3 Seemingly pessimistic update for positive events is misleading

An optimistic update bias for positive stimuli has been described previously (Krieger et al., 2014, Wiswall and Zafar, 2015). Yet Shah et al. (2016), report finding an optimistic asymmetry for negative life events but a pessimistic asymmetry for positive life events. Their studies, however, fall into two methodological pitfalls that need to be guarded against when studying belief updating. We outline these below and then proceed to present the methodology and results of a new study that corrects for these pitfalls. Our new study uses both positive and negative life events but takes care to avoid the pitfalls we outline. Hence it constitutes a valid test of belief updating using both types of life events. We find an optimistic update bias for both negative life events *and* positive life events.

## 2 Methodological pitfall I: Obtaining unreliable, meaningless, statistics for positive events

In the original belief update task (Garrett et al., 2014, Moutsiana et al., 2013, Moutsiana et al., 2015, Sharot et al., 2011, Sharot, Guitart-Masip et al., 2012, Sharot, Kanai et al., 2012), participants are asked to estimate their likelihood of experiencing 80 aversive events in their lifetime (***first estimates***). They are then presented with the likelihood of these events in their population (***information***) and subsequently asked to estimate their likelihoods again (***second estimate***). Trials are then divided into ones where participants received ***good news*** (they learn that an aversive event is less likely than they thought) and trials where participants received ***bad news*** (they learn an aversive event is more likely than they thought). ***Update*** is calculated as the difference between the first and second estimate. When attempting to adapt this task to study positive life events, researchers face potential confounds which if ignored, will lead to invalid conclusions. In particular, whilst validated statistics regarding the likelihood of encountering negative events in one’s life-time are well documented (such as the likelihood of suffering different type of illness or being a victim of crime), statistics about the occurrence of positive life events are not readily available. This is problematic, as the belief update task requires the use of many trials and stimuli. Yet, it is practically impossible to find even 40 positive life events accompanied by validated statistics.

Shah et al. attempt to circumvent this problem by making up statistics for positive events whilst using real, valid, statistics for negative events (see Appendix A of Shah et al., 2016). This systematic asymmetry in the validity of base rates for positive and negative base rates presents a possible confound. For example, for positive events, participants were asked the following questions (try and answer these yourself): How likely are you in your lifetime to: ‘*Attend a friend’s birthday party?*’ ‘*Eat at your favorite restaurant?’ ‘Receive a present?’ ‘Have family visit you at Christmas?’ ‘Being told you are special?’* Most people will have all these events happen to them over a lifetime, thus they are likely to enter estimates close to 100%. This makes it impossible to measure update in a desirable direction (people cannot increase estimates beyond 100%). Participants are likely aware that actual statistics for these questions are less likely to exist than for negative events such as robbery or lung cancer. Note that not all the positive events in Shah et al. are extremely likely, which may explain why mean first estimates reported from their studies are below 50%.

Here, to generate meaningful stimuli for both positive and negative life events we altered the belief update task as follows: we asked participants to estimate their likelihood of encountering everyday life events in the *upcoming month*. First, we obtained the frequencies of such events by asking over 200 participants to report whether different common positive and negative life events occurred to them at least once in the past month. We then used this data to construct a list of base rates for each event (i.e. the likelihood of each life event occurring at least once in a given month in the sample). Next we ran the belief update task on an alternate, but demographically well-matched, set of participants, asking them to estimate their likelihood of encountering these events in the next month.

## 3 Methodological pitfall II: Skewing the distribution of base rate artificially produces a flip for positive events

When investigating biases in belief updating it is important to use a list of base rates that are normally distributed around a mean that sits mid-scale. For example, if a rating scale ranges from 5% to 95% the ideal mean would be 50% and the set of base rates should be normally distributed around this mean. Running simulations we show below that failing to ensure this creates spurious biases in updating (unless one controls for “estimation errors”, see below).

**Fig. 4:**
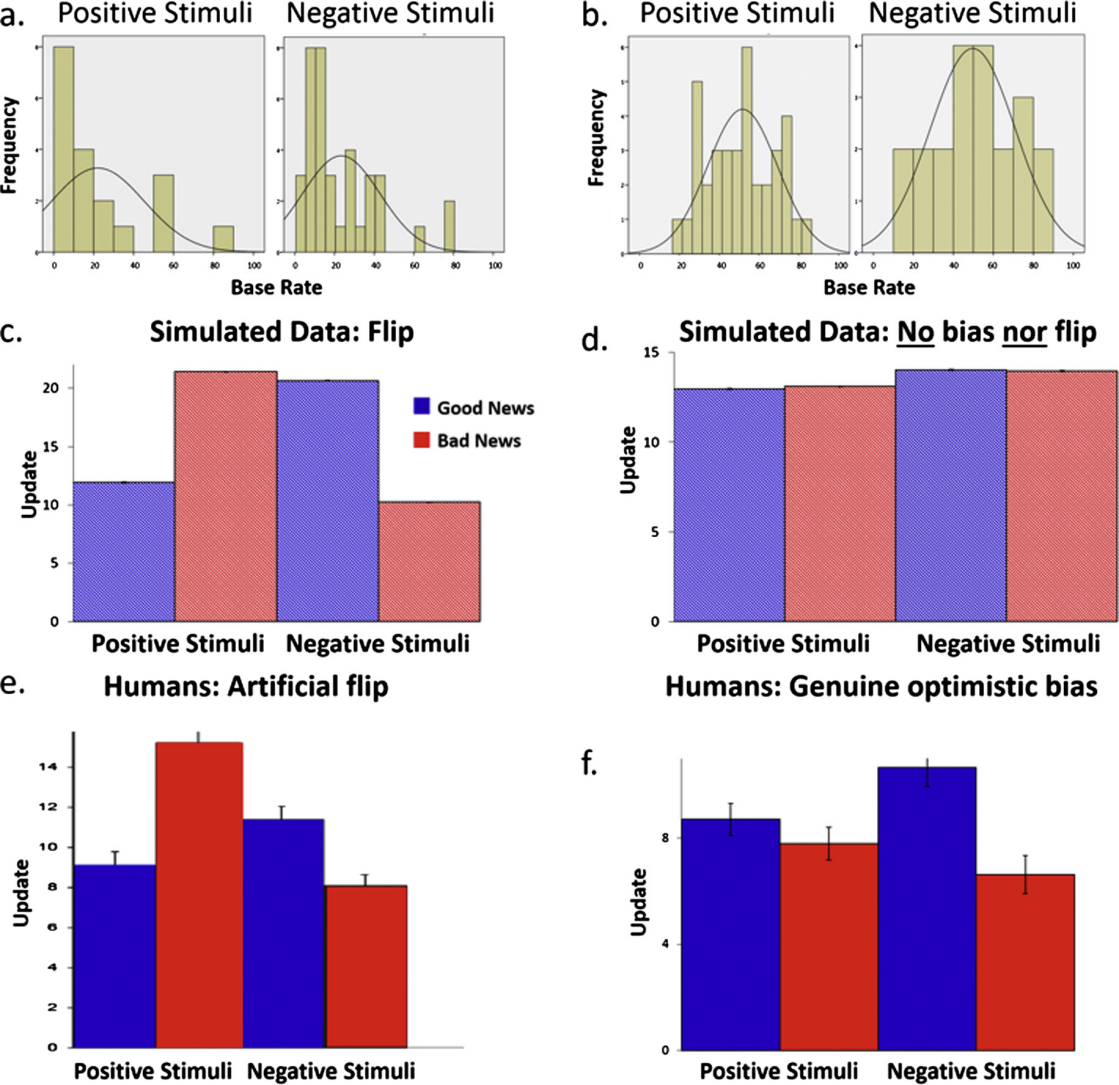
Base rates distributions, simulation results and human data. (a) Most of the base rates included in Shah et al. are probabilities towards the low end of the scale both for positive life events and negative life events. (b) The distribution of base rates used in our new study are normally distributed around the center of the scale used for positive and negative life events. We simulated belief updating using each set of base rates. (c) Using the base rates of Shah et al., simulations reveal an optimistic bias in updating for negative events and a pessimistic update bias for positive events. (e) This pattern closely matches the human data reported by them. (d) Simulations using normally distributed base rates reveal no bias in updating. (f) Human participants nonetheless show an optimistic bias in updating for both positive and negative life events. *Error bars represent SEM*.

Consider four lists of base rates for life events, the distributions of which are shown in Fig. 4a and b. The first two lists are the actual ones used by Shah et al. in two of their experiments (experiments 3A and 3B). The distribution of each set of these base rates is positively skewed (i.e. the majority of base rates are rare and fall below the midpoint of 50%) for positive and negative life events (Fig. 4a). The third and fourth lists are the actual base rates we used in this study. They are normally distributed around 50%, (Fig. 4b).

For each simulation we will randomly generate a ***first estimate*** for each trial for each “participant”. This will be a random integer drawn from a uniform discrete distribution between 0 and 100 for the first two lists of base rates (this is the range of estimates participants are permitted to enter in Shah et al.) or between 5 and 95 for the third and fourth lists of base rates (this is the range of estimates permitted in our experiment). We will then “present” our simulator with the actual base rate for the event on that trial (***information***) and generate a ***second estimate*** - a random integer between the first estimate and the information (also drawn from a uniform distribution). For example, for the question “how likely are you to go out of town for leisure in the upcoming month” our simulator may randomly select 10% (first estimate), it will then observe a base rate of 36% (information) and adjust its answer to a random number between 10 and 36, let’s say 30% (second estimate). Thus, the amount of update on this trial would be 20 (update is calculated such that positive numbers always indicate a move towards the base rate).

We run 1000 such simulations (i.e. “experiments”) for each set of base rates (Fig. 4a and b). If the data produced by a simulation results in biased updating, this will indicate that the bias is due to a statistical artifact (the result of the mathematical constraints of the task) and not to an asymmetry in human learning. If however the simulation produces no bias in updating but a bias is observed for human data, this would suggest that the bias is due to asymmetric learning not to a statistical artifact.

Our simulation clearly shows that when the distribution of base rates is skewed, an artificial bias in belief updating is observed (Fig. 4c). This is not the case however when the base rates are normally distributed (Fig. 4d). Importantly, when an artificial bias is detected it is observed in opposite directions for positive and negative life events creating a distinctive “flip”. Specifically, when base rates are positively skewed (Fig. 4a), the simulation shows greater updating for bad news than good news for positive life events (significant difference in 100% of our simulations, Fig. 4c), whilst for negative life events updating for good news is greater than bad news (significant difference in 100% of our simulations, Fig. 4c). Note that this flip in asymmetry is exactly the pattern reported in Shah et al. (Fig. 4e). When the distribution of base rates is *normally* distributed however, the simulation does not reveal asymmetric updating (Fig. 4d, significant difference in only 5% of cases for positive and negative life events).

Why is an artificial bias produced for skewed distributions? The answer is relatively simple – if the majority of base rates are low numbers then on average there is more room to alter estimates when the first estimate is higher than the base rate, than when it is lower. In other words, the difference between the first estimate and the information given (this is known as the ***estimation error***) will be larger when participants receive bad news for positive events and when they receive good news for negative events. Thus, updates will be greater when information is “bad” for positive events and “good” for negative events, and vice versa when base rates are skewed towards high numbers. This statistical artifact, however, can be corrected by controlling for “estimation errors”. If in the simulations above we control for estimation errors in our analysis no bias is observed for any sets of base rates. This was tested by running an additional 20 simulations (10 for positive life events, 10 for negative life events, 20 participants per simulation) for each set of skewed base rates. For each set of simulated data we then conducted a repeated measures ANOVA with valence (good news/bad news) as a factor and entering the difference in estimation errors between good news and bad news as a covariate. We controlled for estimation errors on a condition level, rather than on every trial, so that we could directly compare our findings to that of Shah et al. When the distribution of base rates was positively skewed (Fig. 4a) a valence effect between good news and bad news was not significant in 19 (95%) of simulations.

Thus, to avoid false conclusions researchers should either use normally distributed base rates with a mean at the midpoint of the scale, control for estimation errors, or do both. In previous studies, estimation errors have either been equal for good news and bad news trials (e.g., Garrett et al., 2014, Sharot et al., 2011, both of which used a scale of 3–77) and/or in instances where differences exist, these are carefully controlled for (e.g., Garrett and Sharot, 2014, Korn et al., 2014). In contrast, whilst the differences between estimation errors in response to desirable and undesirable information can account for the patterns of update observed in Shah et al.’s experiments, estimation errors are not controlled for in their Experiments 1 and 2. In Experiment 3a, when estimation errors are finally controlled for, the critical interaction between valance of stimuli and desirability of information is no longer significant. In Experiment 3b and 4 of Shah et al., it is stated that the results hold when controlling for estimation errors but statistics to support this are not provided.

It is worth noting that the experiments conducted by Shah et al. are not replications of the original belief update studies in design, nor analysis. A few of the differences may have significantly affected the results: (i) out of the 80 negative stimuli used previously, Shah et al. select 38 creating a skewed set. (ii) Shah et al. incentivize participants to remember the base rates provided and require them to input these using a keyboard. This deviation from the original task is important because the novel manipulation may encourage participants to use information in a different manner than they would otherwise. (iii) In contrast to all past studies, Shah et al. *“…exclude trials which were more than 3 interquartile ranges from the mean value of the analyses. These exclusions reduced the total number of trials across the conditions by approximately 2.5%”* (exclusion is reported for Experiments 1, 3a and 3b but not for Experiments 2 and 4). One may assume that this exclusion does not matter, but in at least one documented case the exclusion altered the results from non-significant in a previous version of the Shah et al. manuscript to significant with exclusion of outliers done post hoc. (iv) Different factors are controlled for in each one of the five experiments reported in Shah et al. The reason to control for some factors but not others in the different experiments is not given. We were unable to assess the consequence of this practice, because statistics of the tests after controlling for varied confounding factors were not provided in most instances.

We now proceed to describe a new study, in which we examine how individuals integrate good and bad news into their beliefs about the likelihood of experiencing positive and negative life events, taking care to avoid the two pitfalls we have outlined above.

## 4 Materials and methods

### 4.1 Construction of stimuli

#### 4.1.1 Participants

300 participants located in the United States completed the survey on Mechanical Turk. As in past studies of the belief update task (Garrett and Sharot, 2014, Moutsiana et al., 2013, Moutsiana et al., 2015), we excluded participants with a high Beck Depression Inventory (BDI) score indicating potential depression. 73 participants were excluded for having a BDI score greater than 11 (final sample = 227). Participants were all between the ages of 20 and 30 years of age (inclusive). Completion of the survey took approximately 25 min and participants were compensated for their time.

#### 4.1.2 Task

**Fig. 5:**
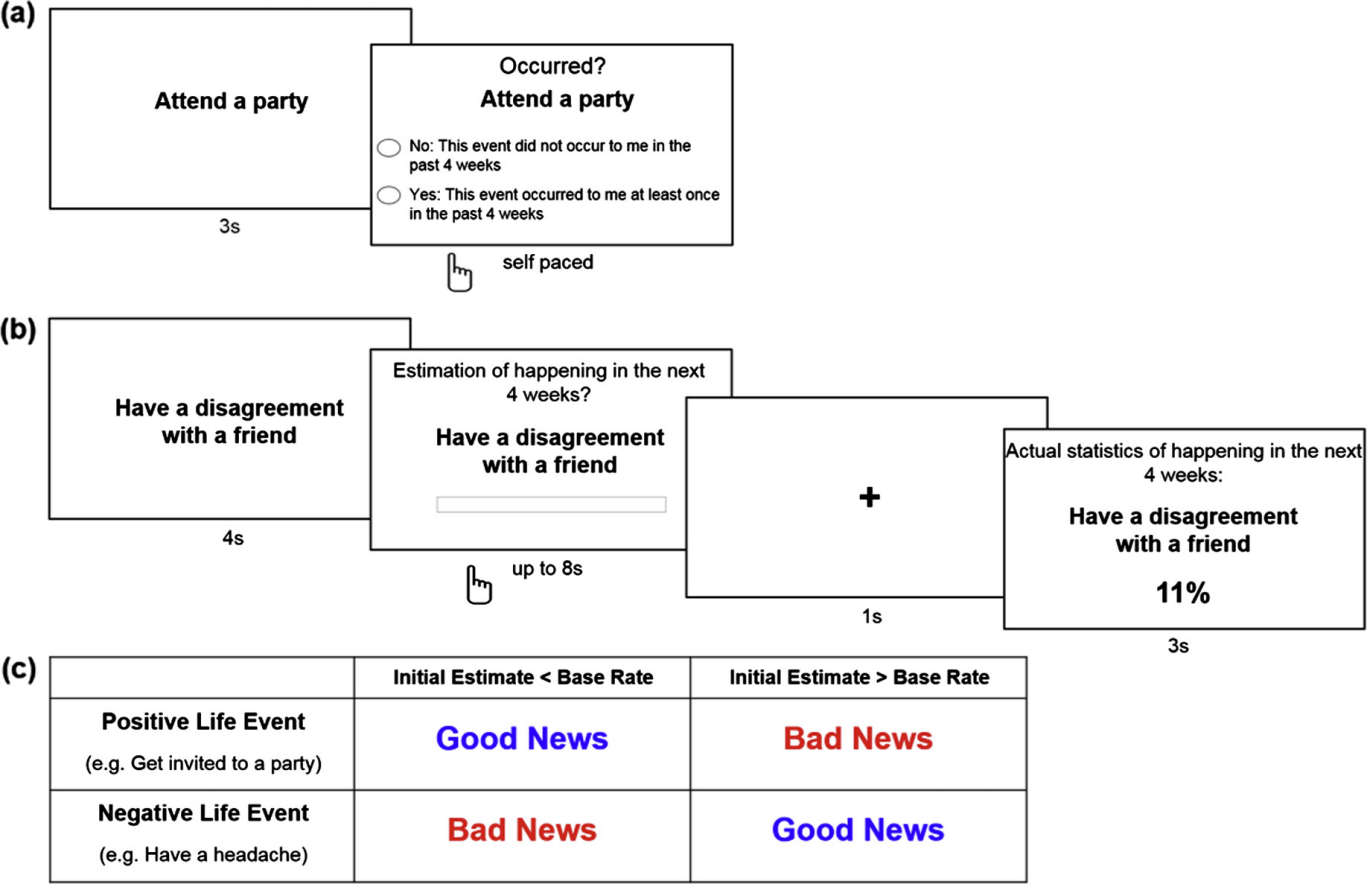
Task design. (a) Construction of stimuli. Participants were presented with 100 commonly occurring positive and negative life events and were instructed to recall whether this event had happened to them in the past 4 weeks. Data was then used to construct a list of base rates. (b) Update Bias Task. On each trial, participants were presented with a short description of 1 of 54 events and asked to estimate how likely this event was to occur to them in the next month. Estimates were entered into a text box displayed on the computer screen using a computer keyboard. They were then presented with the average probability of that event occurring to a person like themselves (calculated from the previous task) in the next month. In a second session (not displayed), participants were asked to re-estimate how likely the event was to occur to themselves. For each event, an update term was calculated as the difference between the participant’s first and second estimations, such that positive numbers indicate a move towards the base rate. (c) Categorization of Events. If participants’ first estimate was lower than the base rate presented, information would be categorized as “good news” if the life event was positive and “bad news” if the life event was negative (column 1). If their first estimate was higher than the base rate presented, the information would be categorized as “bad news” if the event was rated as a positive life event and “good news” if the event was rated as a negative life event (column 2).

The survey began by collecting basic demographic information from participants (age, level of education, marital status, employment status, monthly income) then 2 training examples were presented to familiarize participants with the task. Participants were then presented with 100 different commonly occurring life events for 3 s each. These were a mixture of positive events (for instance: “Discovered a new song you like”, “Laughed at a joke”) and negative events (for instance: “Had an argument with a family member”). Whilst the event was displayed on screen, participants were instructed to recall whether this event had happened to them in the past 4 weeks. They were then asked to indicate either (1) Yes: This event occurred to me at least once in the past 4 weeks; or (2) No: This event did not occur to me in the past 4 weeks. The order of these two options was counterbalanced. Participants had unlimited time to make a response (Fig. 5a).

After completing the survey, participants rated each event on a 5 point likert scale (1 = Very Negative; 2 = Negative; 3 = Neutral; 4 = Positive; 5 = Very Positive) and then completed the BDI (Beck, Ward, Mendelson, Mock, & Erbaugh, 1961) and Life Orientation Test Revised (Scheier, Carver, & Bridges, 1994). The survey was constructed and presented using web based survey service Qualtrics.

#### 4.1.3 Analysis

For each event, the percentage of participants who indicated the event had occurred to them in the past month (out of all participants who completed the study and were included in the final sample) was calculated.

#### 4.1.4 Event selection

A subset of the events (n = 54) were selected for use as stimuli. We selected positive and negative life events such that the distribution of base rates for each type of event (positive and negative) was normally distributed around a mean of 50%. Note that we were unable to verify how accurately participants were able to recall whether events occurred to them or not in the past month. However, since a base rate for a specific event will represent good news to some participants and bad news to others (depending whether a participant overestimates or underestimates the base rate), noise from such inaccuracies ought to cancel out over good news and bad news events in the belief update task.

### 4.2 Belief update task

#### 4.2.1 Participants

200 participants located in the United States (age range 20–30) completed the survey on Mechanical Turk. 56 participants were subsequently excluded for having a BDI score above 11 indicating possible depression. A further 2 participants were excluded because the range of their responses was limited, resulting in zero trials in either the “good news” bin or “bad news” bin, making comparison impossible (final n = 142, mean age: 25.74; mean BDI score: 2.80). There were no differences in age, education, income, marital status or employment status between this set of participants and participants that had completed the base rate survey used to construct the base rate statistics (all P > 0.20). Completion of the survey took approximately 1 h and participants were compensated for their time.

#### 4.2.2 Task

The survey began with an attention check designed to filter out participants that did not read the instructions prudently. Then, demographic information was collected (age, level of education marital status, employment status, monthly income) and 2 training examples provided to familiarize participants with the task.

In the first session, on each trial (54 trials in total) participants were presented with 1 of 54 life events (see Supplementary Material, Table 1 for list of events used) and asked to imagine the event happening to them in the month ahead. They were then asked to estimate how likely that event was to happen to them in the next 4 weeks. Participants were instructed to type in an estimate between 5% and 95% using a computer keyboard. Trials with responses outside this range were excluded from analysis (mean(s.d.) number of responses outside this range: 1.80(3.43)). Participants had 8 s to provide a response. Participants were then shown the base rate statistic of the event happening in the next 4 weeks, which ranged from 15% to 85% (see Fig. 5b). They were told that the statistic was the average likelihood of this event happening at least once in the next four weeks to someone from the same socioeconomic environment as them. In a second session, which took place immediately after the first session, participants were asked to re-estimate how likely each event (54 trials in total) was to happen to them in the next 4 weeks. As in the first session, participants had 8 s to provide a response.

After completion of the task, we tested participants’ memory for the information presented. Participants were asked to recall the information previously presented of each event. Subsequently, participants were then asked to rate all life events according to how positive or negative they found them on a five point Likert scale (1 = very negative, 2 = negative, 3 = neutral, 4 = positive, 5 = very positive). This range of scale was used so that events could be clearly categorized into events that were considered negative (assigned a rating of 1 or 2), neutral (assigned a rating of 3) or positive (assigned a rating of 4 or 5). Participants were also asked to rate past experience with each event (“Has this event happened to you before?” From 1 = never to 6 = very often), as done previously (Garrett and Sharot, 2014, Garrett et al., 2014, Sharot et al., 2011, Sharot, Guitart-Masip et al., 2012, Sharot, Kanai et al., 2012). Three quarters of participants (75%) also rated all events on: vividness (“How vividly could you imagine this event?” From 1 = not vivid to 6 = very vivid); familiarity (“Regardless if this event has happened to you before, how familiar do you feel it is to you from TV, friends, movies and so on?” From 1 = not at all familiar to 6 very familiar); and arousal (“When you imagine this event happening to you how emotionally arousing is the image in your mind?” From 1 = not arousing at all to 6 = very arousing). The scores of these are reported in Supplementary Material, Table 2. Participants then completed the BDI and the Life Orientation Test Revised. The survey was constructed and presented using web based survey service Qualtrics.

#### 4.2.3 Analysis

Life events were categorized as negative or positive for each participant individually according to their own evaluation. Specifically, events were classified as positive if the participant rated the event as 4 (positive) or 5 (very positive) in the ratings section of the task, and negative if rated as a 1 (very negative) or 2 (negative). Events with a neutral rating of 3 were excluded from the analysis (mean(s.d.) number of events with neutral rating: 7.17(5.67)).

For each type of event, participants could receive either “good news” or “bad news” depending on whether the participant initially overestimated or underestimated the probability of the event relative to the base rate (see Fig. 5c). Specifically, if their first estimate was lower than the base rate presented, the information would be categorized as “good news” if the life event was positive and “bad news” if the life event was negative (column 1, Fig. 5c). If their first estimate was higher than the base rate presented, the information would be categorized as “bad news” if the event was rated as a positive life event and “good news” if the event was rated as a negative life event (column 2, Fig. 5c). Trials in which the initial estimate was equal to the statistic presented were excluded from subsequent analyses as these could not be categorized into either condition (less than one negative life event trial and less than one positive life event trial on average per participant).

Belief update was calculated for each trial and participant as the difference between first and second estimate. As done previously (Garrett and Sharot, 2014, Garrett et al., 2014, Moutsiana et al., 2013, Moutsiana et al., 2015, Sharot, Kanai et al., 2012) update was calculated such that positive scores indicate a move towards the base rate, regardless of event type and valence categorization, and negative scores a move away from the base rate.

Update scores were then entered into two general linear models using the IBM SPSS statistics software; one for negative life events and one for positive life events, with information valence as a fixed factor (good news/bad news), participant ID as a random factor and absolute memory errors and absolute estimation errors on each trial as covariates. Since covariates vary from trial to trial, controlling for them on a trial by trial level is more precise than on a condition level as done previously by us (Garrett and Sharot, 2014, Garrett et al., 2014, Moutsiana et al., 2013, Moutsiana et al., 2015, Sharot, Guitart-Masip et al., 2012, Sharot, Kanai et al., 2012) and others (Shah et al., 2016). Nevertheless, to allow direct comparison with Shah et al., we also entered mean update scores for each participant into a 2 (good/bad news) by 2 (positive/negative life event) repeated measures ANOVA controlling for the following covariates on a condition level: (1) the difference in memory for good news trials and bad news trials, both for positive and negative stimuli, (2) the difference in number of good news trials and bad news trials, both for positive and negative stimuli (3) the difference in absolute estimation errors for good news trials and bad news trials, both for positive and negative stimuli (estimation error = |first estimate − base rate|).

## 5 Results

We observed an asymmetry in updating, such that participants updated more in response to good news than bad news. This optimistic update bias was significant both for positive life events (t = −2.88, p < 0.01, mean good news update = 8.71, mean bad news update = 7.79) and for negative life events (t = −4.88, p < 0.001, mean good news update = 10.66, mean bad news update = 6.62). There was also a significant effect of estimation errors (negative events: t = 13.43, p < 0.001; positive: t = 15.81, p < 0.001) and memory errors (negative events: t = −6.13, p < 0.001; positive events: t = −2.92, p < 0.01).

Comparing the bias for positive life events and negative life events by entering mean update scores for each participant into a 2 ∗ 2 repeated measures ANOVA with desirability of information (good/bad news) and life event type (positive/negative life event) as repeated factors (controlling for differences in memory, differences in number of trials and differences in estimation errors) revealed the expected main effect of desirability of information (F(1, 135) = 6.29, p < 0.02), no effect of event type (F(1, 135) = 0.08, p = 0.78) and no interaction (F(1, 135) = 0.31, p = 0.58). Fig. 4f.

## 6 Discussion

The current set of results strongly support a valence dependent asymmetry in how participants update their beliefs, consistent with a large body of fast growing research (Eil and Rao, 2011, Garrett and Sharot, 2014, Garrett et al., 2014, Korn et al., 2012, Krieger et al., 2016, Kuzmanovic et al., 2015, Kuzmanovic et al., 2016, Lefebvre et al., 2016, Ma et al., 2016, Möbius et al., 2012, Sharot, 2011, Sharot and Garrett, 2016, Sharot et al., 2011, Sharot, Guitart-Masip et al., 2012). By pitting this asymmetry against three robustness tests suggested by critics of optimism (Shah et al., 2016) we find that it survives each of these tests, suggesting it is a pervasive phenomenon.

Specifically, we first summarize past data showing that an optimistic update bias exists under a different type of classification (Garrett and Sharot, 2014, Kuzmanovic et al., 2015). Second, we apply the Bayesian analysis outlined by Shah et al. to a previously collected data set (Garrett & Sharot, 2014) and reveal that participants’ updates are more Bayesian in response to good news than bad news. Third, we test Shah et al.’s curious report of a distinctive “flip” in the update asymmetry for positive life events, with updates being greater for bad news compared to good news (the opposite asymmetry to that observed for negative life events). We find that their studies fall into two methodological pitfalls, which can account for their results. Here, we avoid these pitfalls and reveal an optimistic update bias, for both positive life events and negative life events. Moreover, whilst an optimism bias has previously been shown to exist for both everyday and significant life events (Strunk et al., 2006, Weinstein, 1980), biased belief updating had until now only been revealed for the latter (Garrett and Sharot, 2014, Sharot et al., 2011). This is the first demonstration of an optimistic update bias for everyday life events.

The asymmetry in belief updating can result in overly optimistic beliefs. Whilst biased, these beliefs may be adaptive as positive expectations reduce stress and anxiety facilitating physical (Taylor, Kemeny, Reed, Bower, & Gruenewald, 2000) and mental health (Garrett et al., 2014, Korn et al., 2014, Strunk et al., 2006). Furthermore, optimistic expectations enhance motivation and exploration (Bandura, 1989, Puri and Robinson, 2007) increasing the likelihood of gaining resources (Johnson & Fowler, 2011). However, alongside these benefits to optimistic expectations, optimism has also been suggested to reduce necessary precautionary action leading to ill preparedness in the face of natural disasters (Paton, 2003) and failure to adopt preventative measures to safeguard ones health (Shepperd et al., 2013) (see Bortolotti & Antrobus, 2015 for a discussion of the benefits and costs associated with optimistic expectations). On balance it is possible that the positive consequence of unrealistic optimism outweigh the negative, leading humans to evolve this asymmetry in belief formation (McKay & Dennett, 2010).

## Box 1 Comparing participants’ behavior to Bayesian agents.

Following Shah et al. (Section 4.2.4 in Shah et al., 2016), we compare participants update behavior to a rational Bayesian agent by first calculating an implied *Likelihood Ratio*:
*Likelihood Ratio* = *(First Estimate/(*1 − *First Estimate))/(Estimated Base Rate/(*1 − *Estimated Base Rate))*
Note that for this calculation, *First Estimate* and *Estimated Base Rate* are expressed as decimals (e.g., 50% is 0.5). Given this likelihood ratio, we calculate what a Bayesian posterior would be:
*Posterior Odds* *=* *Prior Odds* ∗ *Likelihood Ratio*
Where Prior Odds are given by:
*Prior Odds* *=* *Base rate/(1* *−* *Base Rate)*
Where *Base Rate* is expressed as a decimal.
  Converting Posterior Odds to a percentage is then derived by dividing Posterior Odds by (1 + Posterior Odds). Hence a Bayesian second estimate is given by:
*Bayesian Second Estimate* *=* *Posterior Odds/(1* *+* *Posterior Odds)*
From this, Bayesian Update is calculated for desirable trials as:
*Bayesian Update* *=* *First Estimate* − *Bayesian Second Estimate*
For undesirable trials, Bayesian Update is calculated as:
*Bayesian Update* *=* *Bayesian Second Estimate* − *First Estimate*
For participants, for desirable trials, update is calculated as:
*Participant Update* *=* *First Estimate* − *Second Estimate*
For undesirable trials, Participant Update is calculated as:
*Participant Update* *=* *Second Estimate* − *First Estimate*
Finally, to assess how well participants actual updates correspond to a Bayesian update a difference measure is calculated:
*Difference Measure* *=* *Bayesian Update* − *Participant Update*
Hence larger numbers indicate greater updating for Bayesian agents relative to participants. These scores (difference measure for desirable trials vs difference measure for undesirable trials) are compared using a paired sample *t*-test.

## Footnotes

1 In the latter study, only trials for which classification based on (i) first estimate of own risk, and (ii) estimated base rate were congruent (i.e., both classifications indicated either ‘good news’ or ‘bad news’ were included in the analysis).

2 Readers may misinterpret Shah et al.’s claim regarding the bounded probability scale as simply saying that when a participant receives good news regarding their likelihood of experiencing a negative event (for example that they are less likely to experience robbery than predicted) they would be expected to move down the scale whilst when they receive bad news (for example that they are more likely to experience robbery than predicted) they are expected to move up the scale and this then can create differences in the percentage of movement up and down the scale even when the absolute move is equal. This is not the point that Shah et al. are making. The reason Shah et al. do not attempt to make that point is that in the original paper (Sharot et al., 2011) participants are asked in half the trials how likely they are to experience an event (for example be a victim of card fraud) and in the other half to estimate their likelihood NOT to experience the event (for example never to break a leg). This means that for half the trials, good news will in fact require moving up the scale and bad news will require moving down the scale. The update bias is observed in both of these conditions. It is also observed when update is calculated as a percentage of original estimate (Sharot et al., 2011). The bounded probability claim is made only in relation to Bayesian updating.
