# Supplementary material for: Optimistic update bias holds firm: Three tests of robustness following Shah et al
Source: Conscious Cogn. 2017 Apr;50:12–22. doi: 10.1016/j.concog.2016.10.013 (PMC5380127; doi:10.1016/j.concog.2016.10.013)
Supplement: Supplementary data 1 [file mmc1.docx]

**Supplementary Table 1**

**List of the stimuli used in the study and their respective base rates.**

Base rates were generated from an independent set of participants tasked with reporting whether each event had happened to them at least once in the previous month.

Life events were classified as positive or negative separately for each participant according to their own rating. Hence some events may be classified as positive for some participants but negative for others. There was, however, a high level of agreement among participants (interclass correlation coefficient = 0.75). Life events rated as neutral (i.e. neither positive or negative) were not included in the analysis. On average 27 events were categorized as positive, 18 as negative and 7 as neutral.

| **Life Event** | **Base Rate %** |
| --- | --- |
| *Attend a party* | 45% |
| *Cook dinner for friends* | 36% |
| *Donate money to a needy person or cause* | 37% |
| *50 hours or more sleep in a single week* | 56% |
| *Exercise at least twice in a week* | 70% |
| *Finish reading a book* | 41% |
| *Fix a broken possession* | 39% |
| *Find or receive a gift of a dollar or more* | 56% |
| *Get a haircut* | 45% |
| *Get invited to a party* | 58% |
| *Get taken out for dinner* | 61% |
| *Have a sexual encounter that you enjoy* | 69% |
| *Have a supervisor or teacher praise your work* | 54% |
| *Have an out of town friend visit you* | 30% |
| *Have your photo taken* | 75% |
| *Invite a non-family member to a meal* | 49% |
| *Learn a new skill related to work or school* | 48% |
| *Make a purchase in excess of $50 for your personal enjoyment* | 65% |
| *Meet with your supervisor* | 56% |
| *Participate in a game of sport* | 29% |
| *Play a board game* | 29% |
| *Play with a pet* | 75% |
| *Run into an old friend that you haven’t seen in a long time* | 30% |
| *Receive a pay check* | 81% |
| *Receive a complement about how you dress* | 54% |
| *Shop for clothes* | 56% |
| *Successfully teach someone a new skill or concept* | 50% |
| *Take a day or more of annual leave* | 19% |
| *Try out a new food or dish* | 74% |
| *Try out a new hobby, craft, or sport* | 31% |
| *Go out of town for leisure* | 36% |
| *Wish a friend a happy birthday* | 67% |
| *Win a competitive game of sport* | 22% |
| *Burn something that you are cooking* | 41% |
| *Embarrass yourself* | 60% |
| *Family or friend get ill* | 56% |
| *Find out that someone you know personally has died* | 15% |
| *Get lost* | 26% |
| *Get rejected by someone* | 17% |
| *Get sick or suffered a physical illness* | 41% |
| *Have a disagreement with a friend* | 43% |
| *Have a headache* | 82% |
| *Hear about a natural disaster* | 85% |
| *Hear of a terrorist attack* | 35% |
| *Hurt someone's feelings* | 52% |
| *Ill one day because of overdrinking* | 21% |
| *Received a phone call from a telemarketer* | 52% |
| *Saw a dead animal/human* | 56% |
| *Stay up past 2 AM for school or work* | 40% |
| *Stuck in traffic* | 71% |
| *Teased at/made fun of* | 35% |
| *Get lied to* | 60% |
| *Receive a utility bill* | 78% |
| *Clean the bathroom* | 78% |

**Supplementary Table 2**

Additional ratings provided by 75% of the participants. Prior Experience rated by all participants.

|  | **Positive Life Events, mean (s.d.)** | | **Negative Life Events, mean (s.d.)** | |
| --- | --- | --- | --- | --- |
| **Ratings** | **Good news** | **Bad news** | **Good news** | **Bad news** |
| ***Subjective Scales Questionnaire***  ***(1 = low to 6 = high)*** |  |  |  |  |
| **Familiarity^L, V*L^** | **4.68 (0.77)** | **5.11 (0.70)** | **4.75 (0.84)** | **4.35 (0.99)** |
| **Vividness^L, V*L^** | **4.42 (0.82)** | **4.83 (0.65)** | **4.34 (0.84)** | **3.97 (0.99)** |
| **Emotional arousal^V, L, V*L^** | **3.75 (0.91)** | **3.92 (1.00)** | **3.89 (0.83)** | **3.10 (1.27)** |
| **Prior experience^V, L, V*L^** | **3.80 (0.81)** | **4.51 (0.68)** | **3.75 (0.84)** | **3.18 (0.91)** |

^L^ Main effect *life event* valence (positive/negative) p < 0.05

^V^ Main effect information *valence (good news/bad news)*, p < 0.05

^V*L^ Interaction (valence by life event), p < 0.05

**Supplementary Table 3**

|  | **Positive Life Events, mean (s.d.)** | | **Negative Life Events, mean (s.d.)** | |
| --- | --- | --- | --- | --- |
|  | **Good news** | **Bad news** | **Good news** | **Bad news** |
| **First Estimates** | 29.81 (8.18) | 69.43 (9.09) | 63.02 (11.31) | 28.71 (8.29) |
| **Second Estimates** | 38.51 (11.39) | 61.64 (12.02) | 52.36 (12.78) | 35.33 (12.75) |
| **Base Rates** | 50.65 (5.28) | 48.99 (5.58) | 44.93 (8.64) | 50.39 (6.36) |
| **Estimation Errors** | 20.84 (5.54) | 20.44 (7.12) | 18.09 (6.59) | 21.68 (5.74) |
| **Memory Errors** | 13.71 (5.16) | 14.24 (5.36) | 12.52 (6.40) | 14.47 (5.60) |
